# Multicenter Analytical Performance Evaluation of the BD Phoenix NMIC-461 Panel for Carbapenemase Classification and Antimicrobial Susceptibility Testing of *Enterobacterales*, *Pseudomonas aeruginosa*, and *Acinetobacter* spp.

**DOI:** 10.3390/antibiotics15030286

**Published:** 2026-03-12

**Authors:** Jingjia Zhang, Liying Sun, Ge Zhang, Wei Kang, Tong Wang, Jin Li, Haotian Gao, Qiwen Yang, Kuixia Sun, Qian Wang, Hongli Sun

**Affiliations:** 1 Department of Clinical Laboratory, Peking Union Medical College Hospital, Peking Union Medical College, Chinese Academy of Medical Sciences, Beijing 100730, China; zczjj2009@126.com (J.Z.);; 2 Clinical Biobank, Biomedical Engineering Facility of National Infrastructures for Translational Medicine, Chinese Academy of Medical Sciences, Peking Union Medical College Hospital, Beijing 100730, China; 3 Graduate School, Peking Union Medical College, Chinese Academy of Medical Sciences, Beijing 100730, China; 4 Department of Clinical Laboratory, Peking University First Hospital, Beijing 100034, China; 5 National Clinical Research Center for Laboratory Medicine, Department of Laboratory Medicine, The First Hospital of China Medical University, Shenyang 110001, China

**Keywords:** *Enterobacterales*, *Pseudomonas aeruginosa*, *Acinetobacter* spp., antimicrobial susceptibility testing, carbapenemase, BD Phoenix, NMIC-461

## Abstract

**Objectives:** To evaluate the capability of the BD Phoenix NMIC-461 panel in the detection and classification of carbapenemase production and antimicrobial susceptibility testing of 10 antimicrobial agents among *Enterobacterales*, *Pseudomonas aeruginosa*, and *Acinetobacter* spp. Methods: A total of 714 non-repetitive clinical isolates from three tertiary hospitals in China were enrolled. Carbapenemase production was confirmed by the modified carbapenem inactivation method (mCIM), while carbapenemase typing was validated by polymerase chain reaction (PCR) and Sanger sequencing. Antimicrobial susceptibility testing (AST) for ten antimicrobial agents was performed using broth microdilution (BMD) as the reference method. Results: The sensitivity and specificity of carbapenemase detection were 98.8% (95% CI, 96.6–99.6) and 92.4% (95% CI, 89.5–94.6) separately compared to sequencing. Classification accuracy was compromised by carbapenemase-positive unclassified strains, particularly reducing sensitivity for *Enterobacterales*. Excluding unclassified strains, the sensitivity and specificity were: for class A, 100% (95% CI, 94.0–100) and 97.3% (95% CI, 95.6–98.4); for class B, 97.1% (95% CI, 89.7–99.2) and 97.6% (95% CI, 96.0–98.6); and for class D, 94.0% (95% CI, 87.9–97.3) and 99.1% (95% CI, 97.8–99.7). The panel was subject to limitations for carbapenemase detection when applied to *Pseudomonas aeruginosa*. The NMIC-461 panel demonstrated excellent performance for ten BMD-evaluated agents across four bacterial categories, with essential agreement (EA) exceeding 95% and category agreement (CA) exceeding 90% except for Levofloxacin, and major error (ME) and very major error (VME) rates below 3% and 1.5%, respectively. Conclusions: The BD Phoenix NMIC-461 panel provides reliable AST results for commonly encountered Gram-negative bacterial isolates. Regarding carbapenemase detection, the panel demonstrates high sensitivity but only moderate specificity in classifying carbapenemase-producing organisms (CPO), with a relatively high proportion of positive unclassified isolates among *Enterobacterales* and low specificity for *P. aeruginosa*. Overall, the implementation of NMIC-461 testing holds promise for significantly reducing turnaround time in both carbapenemase detection and classification.

## 1. Introduction

Carbapenemase-producing organisms (CPO) pose a global public health threat. In recent years, carbapenem-resistant *Enterobacterales* (CRE) and non-fermenting drug-resistant bacteria (*Pseudomonas aeruginosa* and *Acinetobacter* spp.) have remained prevalent. CHINET surveillance data indicate that carbapenem-resistant *Klebsiella pneumoniae* (CRKP) increased from 2.9% in 2005 to 25.0% in 2018, while carbapenem-resistant *P. aeruginosa* (CRPA) has shown a downward trend over the past decade, but in 2022, it still accounted for 23.8%, https://www.chinets.com/Data/GermYear (accessed on 28 February 2026). The proportion of carbapenem-resistant *Acinetobacter* spp. increased from 31% in 2005 to 74.5% in 2018, with a slight decrease to 65.8% in 2022 [1]. There are many reasons for the emergence of carbapenem resistance, such as changes or downregulation of porin proteins leading to reduced permeability, or carbapenemase-mediated hydrolysis of carbapenem antibiotics [2,3]. Among these mechanisms, the production of carbapenemase is the most common cause of carbapenem resistance [4]. Furthermore, clinical treatment options vary greatly depending on the carbapenemase types of bacterial strains. Therefore, rapid and accurate detection of the carbapenem-resistant bacterial strains is crucial in controlling the spread of carbapenem-resistant strains, a significant public health concern.

Carbapenemases are classified by the Ambler scheme into three main classes: A, B, and D. Class A carbapenemases, also known as serine carbapenemases (e.g., KPC), are primarily plasmid-mediated and inhibited by avibactam. Class B carbapenemases, also known as metallo-β-lactamases (e.g., NDM, IMP, VIM), are resistant to common β-lactamase inhibitors. Class D mainly comprises OXA-48 and its variants [5,6,7].

The global distribution of CPO has driven innovations in diagnostic technologies and tools to obtain quicker and more accurate results. In clinical microbiology laboratories, the results of antimicrobial susceptibility testing (AST) can provide information on the resistance of bacterial strains to carbapenem antibiotics, suggesting that the strain may produce carbapenemases. Currently, both phenotypic and molecular methods are used to detect the presence of carbapenemases. The most commonly used phenotypic tests include the Carba NP test, modified carbapenem inactivation method (mCIM), and lateral flow immunoassay [8].

In 2024, BD (Becton, Dickinson and Company) introduced the BD Phoenix NMIC-461 panel to China. This panel includes a range of antibiotics, such as imipenem, meropenem, ceftazidime–avibactam, and colistin, in various combinations, and it also has the capability to detect carbapenemases, classifying them according to the Ambler system. Prior to this, countries and regions worldwide had evaluated the performance of the BD Phoenix CPO-related panels using strains isolated within their own territories [7,8,9,10,11,12,13,14].

This multicenter study aimed to evaluate whether the BD Phoenix NMIC-461 panel provides accurate antimicrobial susceptibility testing and reliable carbapenemase classification for clinical isolates in China, thereby potentially serving as an efficient alternative to current methods.

## 2. Results

### 2.1. Isolates Information

**Figure 1:**
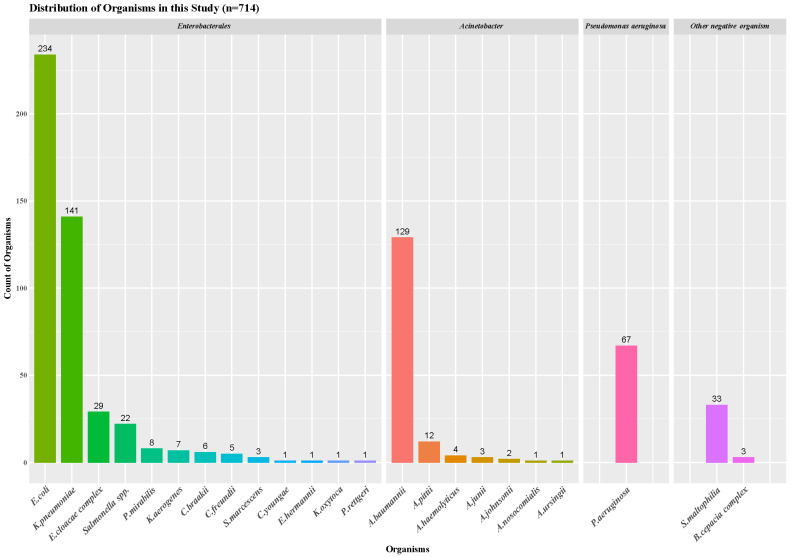
Distribution of organisms in this study (*n* = 714).

In this study, 67 strains of *Pseudomonas aeruginosa*, 152 strains of *Acinetobacter* spp., 459 strains of *Enterobacterales*, and 36 other negative organisms were detected and analyzed. The number of bacteria enrolled in each species is shown in Figure 1.

### 2.2. Performance Evaluation of BD NMIC-461 Detection and Classification of Carbapenemase-Producing Organisms

#### 2.2.1. Performance Evaluation of CPO Detection

A total of 498 bacterial isolates were included in the performance evaluation for the CPO detection, including 433 *Enterobacterales* and 65 *P. aeruginosa*. For the overall 498 isolates, both sensitivity and specificity were above 92%, with a NPV of 99.4% (95% CI, 97.9–99.8); however, the PPV was 83.1% (95% CI, 77.5–87.7). When analyzing *Enterobacterales* and *P. aeruginosa* separately, the *Enterobacterales* demonstrated excellent detection performance, with sensitivity, specificity, PPV, and NPV all exceeding 94%. In contrast, for *P. aeruginosa*, only the NPV was 97.4% (95% CI, 91.2–99.5), while the other three metrics were below 90%, with specificity and PPV as low as 65.5% (95% CI, 52.7–76.4) and 23.1% (95% CI, 13.7–31.6), respectively (Table 1).

#### 2.2.2. Performance Evaluation of CPO Classification

A total of 654 bacterial isolates were included in the performance evaluation for the classification of CPO, consisting of 459 *Enterobacterales*, 129 *Acinetobacter baumannii*, and 66 *P. aeruginosa*. PCR detected 64 strains producing class A enzymes, 68 strains producing class B enzymes, and 117 strains producing class D enzymes (Table 2). Among these, one *P. aeruginosa* strain produced both class A and B enzymes, one *A. baumannii* strain produced both class B and D enzymes, and one *Enterobacter cloacae* strain produced two types of B enzymes (NDM and IMP). The NMIC-461 panel correctly identified only 6 out of the 64 strains producing class A enzymes, with the remaining 58 *K. pneumoniae* detected as positive, unclassified. Of the 68 strains producing class B enzymes, NMIC-461 successfully identified 61, with two strains being misclassified as producing class A and D enzymes, and 5 strains (two *K. pneumoniae*, one *E cloacae*, and one *Citrobacter braakii*) detected as positive unclassified. Among the 117 strains producing class D enzymes, NMIC-461 correctly identified 88, misclassified 3 as producing class B enzymes, 21 positive unclassified *A. baumannii*, and 3 negative *A. baumannii*. Of the 407 PCR-negative strains, NMIC-461 still detected 15, 10, 4, and 2 strains (one *P. aeruginosa* and one *K. pneumoniae*) as producing class A, B, D enzymes and positive unclassified, respectively (Table 2).

To assess the sensitivity, specificity, PPV, and NPV of the NMIC-461 panel in CPO classification, we calculated two sets of data. These calculations were performed by both including and excluding the 86 strains that were detected as positive unclassified by NMIC-461. The resulting data are presented in Table 2.

In the detection of class A β-lactamase, all of the 58 positive unclassified strains were *K. pneumoniae*. As a result, the sensitivity for *Enterobacterales* was only 7.9% (95% CI, 1.3–14.6). If these 58 strains were not included in the analysis, the sensitivity of *Enterobacterales* and all A-class enzyme-producing strains was 100%. The specificity and PPV for *P. aeruginosa* were both relatively low (78.8% (95% CI, 67.0–87.3) and 6.7% (95% CI, 1.2–32.0), respectively), indicating a high number of false positives (13 strains).

In the detection of Class B enzymes, the overall sensitivity was 89.7% (95% CI, 79.9–95.3) (97.1% (95% CI, 89.7–99.2) after excluding 4 positives, unclassified) with a specificity of 97.6% (95% CI, 96.0–98.6), demonstrating robust detection performance. For *P. aeruginosa*, specificity and NPV are 95.1% (95% CI, 86.6–98.3) and 96.7% (95% CI, 88.7–99.1), but the sensitivity and PPV were also relatively low [66.7% (95% CI, 31.0–89.1) and 57.1% (95% CI, 25.1–84.2), respectively], with 2 false negatives (one is positive, unclassified, one is class A). For *Enterobacterales*, sensitivity, specificity, PPV and NPV are 91.8% (95% CI, 81.9–96.6), 98.2% (95% CI, 96.4–99.2), 88.9% (95% CI, 79.0–94.4) and 98.7% (95% CI, 97.1–99.5), 5 false negatives (4 are positive, unclassified, 1 is class D).

In the detection of Class D enzymes, all true positive strains were identified as *A. baumannii.* The specificity, PPV, and NPV were 99.1% (95% CI, 97.8–99.7), 94.7% (95% CI, 88.4–97.7), and 95.0% (95% CI, 92.8–96.6) respectively, while sensitivity was slightly lower at 76.1% (95% CI, 67.2–83.2) (94.0% (95% CI, 87.9–97.3) after excluding 21 unclassified positive strains). NMIC-461 detected 28 false negatives (including 21 positives, unclassified, 4 class B, and 3 negatives), leading to relatively low sensitivity and NPV [76.1% (95% CI, 67.2–83.2) and 53.5% (95% CI, 41.3–65.0)], respectively, of *A. baumannii*. Additionally, one false positive (sequencing as class B) was identified among the *Enterobacterales*, and three false positives were detected in *P. aeruginosa*.

Overall, from the comprehensive evaluation of NMIC-461 for detecting CPO, sensitivity, specificity, and NPV all exceeded 90%, with PPV being slightly lower at 88.8% (95% CI, 84.5–92.1). However, certain limitations requiring further optimization were identified; the main issue with NMIC-461 in the detection of CPO was the high number of positive unclassified results, which accounted for 34.8% of all CPO positive strains (86/247). A total of 31 false positives were detected, including 20 cases of *P aeruginosa*, 10 cases in *Enterobacterales*, and 1 case of *A. baumannii*.

### 2.3. Performance of BD NMIC-461 AST Results

The provisions in CLSI M52 [15] were used as the criteria for the evaluation of CA, EA, MIE, ME, and VME of strains (Table 3). Overall, the NMIC-461 system demonstrates excellent performance in antimicrobial susceptibility testing for ten antibiotics across four bacterial categories. With EA exceeding 95% and CA exceeding 90% except for levofloxacin, ME and VME remain below 3% and 1.5%, respectively. These metrics indicate high accuracy and reliability in detecting antimicrobial susceptibility profiles.

Among the ten antibiotics evaluated, ceftazidime-avibactam and colistin—two key agents in clinical CPO management—showed particularly strong performance with NMIC-461. The system demonstrated 100% CA for ceftazidime-avibactam susceptibility testing against both *Enterobacterales* and *P. aeruginosa*, with EA rates of 97% and 100%, respectively. No ME or VME was detected for this antibiotic. Similarly, colistin testing-maintained CA and EA values exceeding 95% across *Enterobacterales*, *P. aeruginosa*, and *Acinetobacter* spp., with no VME observed during validation.

The ME rate for the detection of *Acinetobacter* spp. by ceftazidime was 4.8% (1/21). This observation may be attributed to the limited number of *Acinetobacter* spp. strains included in the study cohort. Nevertheless, the system-maintained CA and EA values exceeding 96%, with zero VME detected throughout the testing process. The CA rate for the detection of *Enterobacterales* by ciprofloxacin was somewhat reduced at 88.5% (376/425). The MIE rate of 11.5% was primarily attributed to excessive intermediate categorization, which compromised CA. Notably, EA remained robust at 97.9%, demonstrating preserved methodological accuracy despite interpretive challenges. For the detection of *P. aeruginosa* by imipenem, the ME rate was relatively high at 11.8% (2/17), and the CA rate was low at 83.3% (20/24). The comparative distributions of MICs for various bacterial strains, as determined by the NMIC-461 panel and the broth microdilution (BMD) method, are presented in Figures S2–S5.

## 3. Discussion

In this study, a total of 714 non-repetitive clinical isolates were initially enrolled. However, during the analysis of antimicrobial susceptibility or enzyme production capability, isolates that exhibited poor growth, no growth on the panel, or contamination were excluded from the corresponding statistical evaluations. As a result, the number of isolates included in the analysis varied across different antimicrobial agents. Detailed exclusion criteria and the corresponding numbers of excluded isolates are provided in Text S1.

In China, the rising isolation rate of CPOs has led to constrained therapeutic options. Current clinical management of CPO infections primarily relies on ceftazidime/avibactam, polymyxin B, tigecycline, and carbapenem-β-lactamase inhibitor combinations. As the CPO detection and classification results of this panel are reported at the same time with the above antibiotic drugs, compared to routine testing, the implementation of the CPO test allowed a mean reduction of 21.3 h (95% CI, 17.6–25) in turnaround time, 16.8 min (95% CI, 13.4–20.2) in hands-on time, and 20.6 CHF (95% CI, 16.5–24.8) in costs [16].

Notably, carbapenems such as imipenem remain empirically prescribed for Gram-negative bacterial infections prior to CPO confirmation through microbiological identification. Overall, the NMIC-461 demonstrated robust performance in AST for imipenem, with CA and EA both exceeding 95%, while ME and VME rates were maintained below 0.6%. The assay exhibited strong concordance for *Enterobacterales* and *Acinetobacter* spp. However, its performance was less robust for *P. aeruginosa* isolates. The ME rate was 11.8% (2/17), with both CA and EA falling below 90%. Previous studies have also reported low CA values for imipenem detection using BD panels [17]. Imipenem is typically degraded by dehydropeptidase-1 (DHP-1), necessitating co-administration with a DHP-1 inhibitor, such as cilastatin [16]. This characteristic of imipenem may contribute to the observed challenges in its susceptibility testing performance.

In the detection and classification of CPOs, NMIC-461 yielded a certain number of positive unclassified results, primarily involving 58 *K. pneumoniae* strains producing KPC and 21 *A. baumannii* strains producing OXA. However, in clinical isolates commonly encountered in China, Class A and Class D carbapenemase-producing strains are predominantly *K. pneumoniae* and *A. baumannii*, respectively [18,19]. Chinese antimicrobial-resistant bacterial strains share clonal dissemination with those from other countries/regions, yet exhibit distinct strain predominance patterns [20]. For instance, carbapenem-resistant *K. pneumoniae* (CRKP) predominantly exhibits sequence type ST11 in China, contrasting with the ST258 dominance observed in Europe and the United States [21].

Previous international studies on BD CPO panels from multiple countries have consistently detected positive unclassified strains harboring diverse resistance mechanisms [9,10,11,12,13,14], including various carbapenemase types, ESBLs, AmpC enzymes, and potentially novel uncharacterized carbapenemase resistance determinants. Phenotypic detection and classification of such strains remain challenging due to co-occurring resistance mechanisms that interfere with antimicrobial agent/β-lactamase inhibitor combination-based phenotypic assays [14]. Despite the number of unclassified positive results, NMIC-461 can still provide clinicians with a preliminary and actionable reference result in a timely manner.

NMIC-461 exhibited a higher rate of false positives in both the detection and classification of carbapenemase production in *P. aeruginosa*. This phenomenon may be attributed to the intrinsic or acquired resistance of *P. aeruginosa* to nearly all available antimicrobial agents. Multiple mechanisms can contribute to its antimicrobial resistance, including the production of carbapenemases, target site mutations, loss of outer membrane proteins, and multidrug efflux systems [9]. Resistance to carbapenems in *P. aeruginosa* is primarily mediated by non-carbapenemase mechanisms, such as the loss of OprD porin expression and/or upregulation of the MexAB-OprM efflux pump. The specificity of NMIC-461 may be compromised by the non-specific effects of inhibitors used in the assay, which can disrupt bacterial outer membrane permeability through chelation. Additionally, low carbapenem hydrolysis activity, lack of or minimal expression of carbapenemase genes, and slow bacterial growth could also contribute to the failure of NMIC-461 detection [12]. The reference method used to define which carbapenemases are present is limited to a specific set of genes, so some apparent false positives or positive unclassified results might actually be caused by other enzymes or resistance mechanisms that were not tested. Given these limitations, it is recommended that positive results be confirmed using an alternative method for *P. aeruginosa*.

In this study, we enrolled a sufficient number of isolates through a multicenter approach, particularly for *Enterobacterales*. In comparison with previous international studies of a similar nature, we revealed a higher incidence of positive unclassified results. Notably, our study demonstrated a low false-negative rate. After excluding positive unclassified strains, only two and seven false negatives were identified in class B and class D carbapenemase detection assays, respectively. However, false positives were observed in the detection of Class A, B, and D carbapenemases. Chiou et al. [7] evaluated the classification performance of Carba NP, mCIM, and BD Phoenix CPO in detecting carbapenemase production among 190 *Enterobacterales* isolates. The sensitivity and specificity of BD Phoenix CPO were 89.4% and 66.7%, respectively, while the other two methods achieved values above 90%. Similarly, Cho et al. [22] tested 47 carbapenemase-producing *Enterobacterales* (CPE), 52 carbapenem-resistant *Enterobacterales* without carbapenemase production (non-CP-CRE), and 136 carbapenem-susceptible *Enterobacterales* (CSE). The sensitivity and specificity for detecting CPOs were 97.9% and 100%, respectively, whereas the specificity for non-CP-CREs was only 32.7%. These findings also highlight the issue of significant false positives in carbapenemase detection assays.

In summary, the BD NMIC-461 panel can provide relatively reliable antimicrobial susceptibility results for commonly encountered Gram-negative bacterial isolates. BD NMIC-461 is capable of detecting and classifying CPO and integrates this functionality into routine antimicrobial susceptibility testing, which potentially reduces the turnaround time for CPO detection. The strains with unclassified positive results could be confirmed by other alternative methods and could refer to the related antibiotic agents’ results reported at the same time by the same panel. Clinicians can integrate MIC values of carbapenems, ceftazidime-avibactam, and colistin from the panel to inform therapeutic decisions (Table S2). Overall, implementation of the Phoenix NMIC-461 panel may positively influence laboratory workflow optimization and enhance evidence-based therapeutic and infection control strategies.

## 4. Materials and Methods

### 4.1. Isolates

In this study, we enrolled 714 non-repetitive clinical isolates from three tertiary hospitals in China, including 459 *Enterobacterales*, 152 *Acinetobacter* spp., 67 *P. aeruginosa*, and 36 other Gram-negative bacteria. The isolates were inoculated into cryovials containing 20% (*w*/*v*) skimmed milk and stored at −80 °C. From July 2020 to October 2022, the performance of the BD Phoenix NMIC-461 panel was evaluated at the three centers. The study was approved by the Research Ethics Committee of Peking Union Medical College Hospital (Ethical Approval Number: KS2021282).

### 4.2. BD Phoenix

The BD Phoenix NMIC-461 panel includes 10 antimicrobial agents that were evaluated, which include Amoxicillin-Clavulanic Acid, Ceftaroline, Ceftazidime-Avibactam, Ceftazidime, Ceftriaxone, Ciprofloxacin, Colistin, Imipenem, Levofloxacin, and Meropenem. The BMD method served as the reference method. The detection and classification of carbapenemase-producing organisms in this panel was evaluated compared to mCIM and sequencing.

Antimicrobial susceptibility testing is conducted according to the manufacturer’s instructions. A 0.5–0.6 McFarland turbidity standard bacterial suspension is prepared using the Phoenix turbidity meter. A 25 µL bacterial suspension is transferred into Phoenix AST broth, and before adding it to the plates, one drop of Phoenix AST indicator is added to detect microbial growth. The panel is sealed, recorded, and loaded into the Phoenix M50 system. After incubation for 16 h, the results are interpreted using the Epicenter Data Management Software, Version 7.22A (BD Diagnostics, Franklin Lakes, NJ, USA) [17].

### 4.3. Broth Microdilution (BMD)

The standard BMD method is performed according to the guidance document ISO 20776-1 [23]. *E. coli* ATCC 25922, *P. aeruginosa* ATCC 27853, *K. pneumoniae* ATCC 700603, and *K. pneumoniae* ATCC BAA-1705 were used as quality control strains as recommended by Clinical and Laboratory Standards Institute (CLSI) M100, 2023 [24].

### 4.4. Modified Carbapenem Inactivation Method (mCIM)

In this study, mCIM was used as the control method for carbapenemase production in isolates. mCIM was performed according to the CLSI guidelines to detect the presence of carbapenemase [24]. For each isolate tested, a 1 μL loopful of bacteria for *Enterobacterales* or a 10 μL loopful of bacteria for *Pseudomonas aeruginosa* from an overnight blood agar plate was emulsified in 2 mL Trypticase Soy Broth (TSB). A 10 µg meropenem disk was placed in each tube, and the tubes were incubated at 35 °C for 4 h ± 15 min. Subsequently, the disks were removed and applied to MH agar plates (Oxoid, Basingstoke, UK) freshly plated with a 0.5 McFarland suspension of a carbapenem-susceptible *E. coli* ATCC 25922 strain. The plates were incubated at 35 °C for 16–20 h. The mCIM result was considered to be carbapenemase negative if the zone diameter was ≥19 mm, positive if the zone diameter was 6–15 mm, or the presence of pinpoint colonies within a 16–18 mm zone. The presence or absence of a carbapenemase could not be confirmed if the zone diameter was 16–18 mm or ≥19 mm, and the presence of pinpoint colonies within the zone.

### 4.5. Screening of Carbapenemase Genes

All enrolled *Enterobacterales*, *P. aeruginosa*, and *Acinetobacter* spp. were Sanger sequenced, and Ambler classified to determine whether the enzyme class detected by the NMIC-461 plate was accurate. The polymerase chain reaction (PCR) and Sanger sequencing were employed to screen out carbapenemase genes, including KPC, NDM, VIM, IMP, OXA-48, OXA-23, OXA-24, and OXA-58. The oligonucleotide sequences of the primers are listed in Supplementary Table S1. The PCR products were sequenced and analyzed using BLAST (Version 2.17.0), http://www.ncbi.nlm.nih.gov/BLAST, (accessed on 28 February 2026). The BLAST cut-off was a percentage identity of 100%.

### 4.6. Data Analysis

Sensitivity, specificity, positive predictive value (PPV), and negative predictive value (NPV) were calculated to assess the ability of NMIC-461 to detect carbapenemase-producing compared to mCIM and carbapenemase genes. The same parameters were also used to assess the ability of NMIC-461 to detect enzyme-producing Ambler classification of strains. Agreement and validity values were calculated with a 95% confidence interval (CI) based on an exact binomial distribution. For the drugs with BMD as the control method, essential agreement (EA), category agreement (CA), minor error (MIE), major error (ME), and very major error (VME) were calculated separately [15]. All analyses were performed using R Statistical Software (v4.4.0; R Core Team 2024).

## Figures and Tables

**Table 1:** NMIC-461 of the CPO results and agreement results summary compared to the mCIM method.

Organism	NMIC-461 Result	mCIM Result						
		Positive, *n* (%)	Negative, *n* (%)	Total, *n* (%)	Sensitivity, % (95% CI)	Specificity, % (95% CI)	PPV, % (95% CI)	NPV, % (95% CI)
Total	Positive	133 (98.5)	27 (7.4)	160 (32.1)	98.5 (94.8–99.6)	92.6 (89.4–94.8)	83.1 (77.5–87.7)	99.4 (97.9–99.8)
	Negative	2 (1.5)	336 (92.6)	338 (67.9)				
	Total	135 (100)	363 (100)	498 (100)				
*Enterobacterales*	Positive	127 (99.2)	7 (2.3)	134 (30.9)	99.2 (95.7–99.9)	97.7 (95.3–98.9)	94.8 (89.9–97.4)	99.7 (98.2–99.9)
	Negative	1 (0.8)	298 (97.7)	299 (69.1)				
	Total	128 (100)	305 (100)	433 (100)				
*Pseudomonas aeruginosa*	Positive	6 (85.7)	20 (34.5)	26 (40)	85.7 (48.7–97.4)	65.5 (52.7–76.4)	23.1 (13.7–31.6)	97.4 (91.2–99.5)
	Negative	1 (14.3)	38 (65.5)	39 (60)				
	Total	7 (100)	58 (100)	65 (100)				

**Table 2:** NMIC-461 of the CPO subgroup results and agreement results summary compared to the PCR sequencing method.

Organism	PCR Results		NMIC_461 Results							Sensitivity, % (95% CI)	Specificity, % (95% CI)	PPV, % (95% CI)	NPV, % (95% CI)	Kappa (95% CI)
			Positive					Negative, *n*	Total, *n*					
			A, *n*	B, *n*	D, *n*	Positive Unclassified, *n*	Total, *n*							
All CPOs	Positive	A	6	0	0	58	64	0	64	9.4 (4.4–18.8) ^d^ 100 (94.0–100) *	97.3 (95.6–98.4) ^d^	27.3 (12.9–48.1) ^d^ 80 (69.7–87.6) *	90.9 (88.3–92.9) ^d^ 100 (99.3–100) *	0.09 (0.01–0.18) ^d^ 0.875 (0.815–0.936) *
		B	1	61	1	5	68	0	68	89.7 (79.9–95.3) ^e^ 97.1 (89.7–99.2) *	97.6 (96.0–98.6) ^e^	81.3 (71.3–88.5) ^e^ 82.5 (72.8–89.3) *	98.8 (97.5–99.5) ^e^ 99.7 (98.6–99.9) *	0.888 (0.832–0.945) ^e^ 0.807 (0.739–0.876) *
		D	0	4	89	21	114	3	117	76.1 (67.2–83.2) ^f^ 94.0 (87.9–97.3) *	99.1 (97.8–99.7) ^f^ 99.1 (97.8–99.7) *	94.7 (88.4–97.7) ^f^ 95.7 (90.2–98.2) *	95.0 (92.8–96.6) ^f^ 98.7 (97.3–99.4) *	0.807 (0.739–0.876) ^f^ 0.942 (0.904–0.979) *
	Negative		15	10	4	2	31	376	407	-				
	Total		22	75	94	86	277	379	656 ^a^	98.8 (96.6–99.6) ^g^	92.4 (89.5–94.6) ^g^	88.8 (84.5–92.1) ^g^	99.2 (97.8–99.8) ^g^	0.909 (0.874–0.944) ^g^
*Acinetobacter baumannii*	Positive	A	0	0	0	0	0	0	0	-	100 (100–100) ^d^	-	100 (100–100) ^d^	–
		B	0	1	0	0	1	0	1	100 (20.7–100) ^e^	96.9 (92.3–98.9) ^e^	20.0 (3.6–62.5) ^e^	100 (97.2–100) ^e^	0.305 (0.014–0.595) ^e^
		D	0	4	89	21	114	3	117	76.1 (67.2–83.2) ^f^ 94.0 (87.9–97.3) *	97.0 (84.2–99.6) ^f^ 97.0 (84.2–99.6) *	98.9 (94.1–99.8) ^f^ 99.1 (95.1–99.9) *	53.5 (41.3–65.0) ^f^ 82.1 (67.3–91.1) *	0.523 (0.406–0.641) ^f^ 0.848 (0.749–0.947) *
	Negative		0	0	1	0	1	11	12	-				
	Total		0	5	90	21	116	14	130 ^b^	97.5 (92.8–99.2) ^g^	91.7 (61.5–98.4) ^g^	99.1 (95.3–99.9) ^g^	78.6 (52.4–92.4) ^g^	0.834 (0.671–0.997) ^g^
*Enterobacterales*	Positive	A	5	0	0	58	63	0	63	7.9 (1.3–14.6) ^d^ 100 (94.2–100) *	99.5 (98.2–99.9) ^d^	71.4 (35.9–91.8) ^d^ 96.9 (89.3–99.2) *	87.2 (83.7–90.0) ^d^ 100 (99.0–100) *	0.125 (0.041–0.209) ^d^ 0.978 (0.949–1.000) *
		B	0	56	1	4	61	0	61	91.8 (81.9–96.6) ^e^ 98.4 (91.1–99.8) *	98.2 (96.4–99.2) ^e^	88.9 (79.0–94.4) ^e^ 89.6 (79.9–95.0) *	98.7 (97.1–99.5) ^e^ 99.7 (98.6–99.9) *	0.894 (0.836–0.953) ^e^ 0.936 (0.886–0.987) *
		D	0	0	0	0	0	0	0	-	99.8 (98.9–99.99) ^f^	0 (0.0–79.4) ^f^	100 (99.2–100) ^f^	–
	Negative		2	7	0	1	10	325	335	-				
	Total		7	63	1	63	134	325	459	100 (97.0–100) ^g^	97.0 (94.6–98.5) ^g^	92.5 (86.9–96.0) ^g^	100 (98.9–100) ^g^	0.95 (0.919–0.981) ^g^
*Pseudomonas aeruginosa*	Positive	A	1	0	0	0	1	0	1	100 (20.7–100.0) ^d^	78.8 (67.0–87.3) ^d^	6.7 (1.2–32.0) ^d^	100 (93.1–100) ^d^	0.105 (0.008–0.201) ^d^
		B	1	4	0	1	6	0	6	66.7 (31.0–89.1) ^e^ 83.3 (43.7–97.0) *	95.1 (86.6–98.3) ^e^	57.1 (25.1–84.2) ^e^ 62.5 (30.6–86.3) *	96.7 (88.7–99.1) ^e^ 98.3 (90.9–99.8) *	0.605 (0.340–0.871) ^e^ 0.727 (0.490–0.964) *
		D	0	0	0	0	0	0	0	-	95.5 (87.5–98.6) ^f^	0 (0.0–56.2) ^f^	100 (94.2–100) ^f^	–
	Negative		13	3	3	1	20	40	60	-				
	Total		15	7	3	2	27	40	67 ^c^	100 (64.6–100.0) ^g^	66.7 (53.7–77.6) ^g^	25.9 (13.5–44.4) ^g^	100 (91.2–100) ^g^	0.29 (0.147–0.432) ^g^

^a,b,c^: The PCR sequencing enzyme types of one *Pseudomonas aeruginosa* are Class A and B, and are treated as PCR positive simultaneously when counting in Class A and B. The PCR sequencing enzyme types of one *Acinetobacter baumannii* are Class B and D, and are treated as PCR positive when counting in Class B and D simultaneously. Therefore, when counting all CPOs, the number will be 2 more than the total number of enrolled strains (654 strains). Similarly, when counting *Acinetobacter baumannii* and *Pseudomonas aeruginosa*, the numbers will be 1 more than the respective numbers of enrolled strains (129 strains and 66 strains), respectively. ^d^: The data in this part are respectively the sensitivity, specificity, PPV, and NPV of Class A enzymes. The sensitivity, specificity, PPV, and NPV without asterisks represent the results of the strains that were included as NMIC-461 test positive unclassified; ^e^: The data in this part are respectively the sensitivity, specificity, PPV, and NPV of Class B enzymes. The sensitivity, specificity, PPV, and NPV without asterisks represent the results of the strains that were included as NMIC-461 test positive unclassified. ^f^: The data in this part are respectively the sensitivity, specificity, PPV, and NPV of Class D enzymes. The sensitivity, specificity, PPV, and NPV without asterisks represent the results of the strains that were included as NMIC-461 test positive unclassified. ^g^: The data in this part are respectively the sensitivity, specificity, PPV, and NPV of all CPOs, *Acinetobacter baumannii*, *Enterobacterales*, and *Pseudomonas aeruginosa* for the detection of carbapenemases. *: The sensitivity, specificity, PPV, and NPV with asterisks represent the results after excluding the strains that were NMIC-461 test positive unclassified.

**Table 3:** NMIC-461 of SIR and agreement results summary compared to BMD.

Antimicrobial Agent	Organism	BMD SIR Result			NMIC_461 Agreement Result Compared to BMD				
		S, *n* (%)	I, *n* (%)	R, *n* (%)	EA, % (95% CI)	CA, % (95% CI)	MIE, % (95% CI)	ME, % (95% CI)	VME, % (95% CI)
Amoxicillin-Clavulanate	Total	230 (53.7%)	40 (9.3%)	158 (36.9%)	98.8% (97.3%, 99.5%)	93.5% (90.7%, 95.4%)	6.5% (4.6%, 9.3%)	0.0% (0.0%, 1.6%)	0.0% (0.0%, 2.4%)
	*Enterobacterales*	230 (53.7%)	40 (9.3%)	158 (36.9%)	98.8% (97.3%, 99.5%)	93.5% (90.7%, 95.4%)	6.5% (4.6%, 9.3%)	0.0% (0.0%, 1.6%)	0.0% (0.0%, 2.4%)
	*Acinetobacter*	not tested	not tested	not tested					
	*Pseudomonas aeruginosa*	not tested	not tested	not tested					
	Other negative organisms	not tested	not tested	not tested					
Ceftaroline	Total	176 (41.9%)	5 (1.2%)	239 (56.9%)	99.8% (98.7%, 100.0%)	98.1% (96.3%, 99.0%)	1.7% (0.8%, 3.4%)	0.6% (0.1%, 3.1%)	0.0% (0.0%, 1.6%)
	*Enterobacterales*	176 (41.9%)	5 (1.2%)	239 (56.9%)	99.8% (98.7%, 100.0%)	98.1% (96.3%, 99.0%)	1.7% (0.8%, 3.4%)	0.6% (0.1%, 3.1%)	0.0% (0.0%, 1.6%)
	*Acinetobacter*	not tested	not tested	not tested					
	*Pseudomonas aeruginosa*	not tested	not tested	not tested					
	Other negative organisms	not tested	not tested	not tested					
Ceftazidime-Avibactam	Total	395 (85.5%)	0 (0%)	67 (14.5%)	97.2% (95.2%, 98.4%)	100.0% (99.2%, 100.0%)	0.0% (0.0%, 0.8%)	0.0% (0.0%, 1.0%)	0.0% (0.0%, 5.4%)
	*Enterobacterales*	366 (85.5%)	0 (0%)	62 (14.5%)	97.0% (94.9%, 98.2%)	100.0% (99.1%, 100.0%)	0.0% (0.0%, 0.9%)	0.0% (0.0%, 1.0%)	0.0% (0.0%, 5.8%)
	*Acinetobacter*	not tested	not tested	not tested					
	*Pseudomonas aeruginosa*	29 (85.3%)	0 (0%)	5 (14.7%)	100.0% (89.8%, 100.0%)	100.0% (89.8%, 100.0%)	0.0% (0.0%, 10.2%)	0.0% (0.0%, 11.7%)	0.0% (0.0%, 43.4%)
	Other negative organisms	not tested	not tested	not tested					
Ceftazidime ^1^	Total	251 (44.7%)	23 (4.1%)	287 (51.2%)	98.4% (97.0%, 99.2%)	94.7% (92.5%, 96.2%)	4.6% (3.2%, 6.7%)	0.4% (0.1%, 2.2%)	1.0% (0.4%, 3.0%)
	*Enterobacterales*	198 (50.8%)	19 (4.9%)	173 (44.4%)	98.7% (97.0%, 99.4%)	95.1% (92.5%, 96.9%)	4.4% (2.7%, 6.9%)	0.0% (0.0%, 1.9%)	1.2% (0.3%, 4.1%)
	*Acinetobacter*	21 (18.6%)	1 (0.9%)	91 (80.5%)	98.2% (93.8%, 99.5%)	96.5% (91.2%, 98.6%)	2.7% (0.9%, 7.5%)	4.8% (0.8%, 22.7%)	0.0% (0.0%, 4.0%)
	*Pseudomonas aeruginosa*	18 (78.3%)	1 (4.3%)	4 (17.4%)	100.0% (85.7%, 100.0%)	95.7% (79.0%, 99.2%)	4.3% (0.8%, 21.0%)	0.0% (0.0%, 17.6%)	0.0% (0.0%, 49.0%)
	Other negative organisms	14 (40%)	2 (5.7%)	19 (54.3%)	94.3% (81.4%, 98.4%)	82.9% (67.3%, 91.9%)	14.3% (6.3%, 29.4%)	0.0% (0.0%, 21.5%)	5.3% (0.9%, 24.6%)
Ceftriaxone ^1^	Total	189 (43.9%)	1 (0.2%)	241 (55.9%)	99.5% (98.3%, 99.9%)	99.5% (98.3%, 99.9%)	0.2% (0.0%, 1.3%)	0.0% (0.0%, 2.0%)	0.4% (0.1%, 2.3%)
	*Enterobacterales*	186 (43.5%)	1 (0.2%)	241 (56.3%)	99.5% (98.3%, 99.9%)	99.5% (98.3%, 99.9%)	0.2% (0.0%, 1.3%)	0.0% (0.0%, 2.0%)	0.4% (0.1%, 2.3%)
	*Acinetobacter*	3 (100%)	0 (0%)	0 (0%)	100.0% (43.8%, 100.0%)	100.0% (43.8%, 100.0%)	0.0% (0.0%, 56.2%)	0.0% (0.0%, 56.2%)	
	*Pseudomonas aeruginosa*	not tested	not tested	not tested					
	Other negative organisms	not tested	not tested	not tested					
Ciprofloxacin	Total	184 (32.2%)	38 (6.6%)	350 (61.2%)	97.0% (95.3%, 98.1%)	90.7% (88.1%, 92.8%)	9.3% (7.2%, 11.9%)	0.0% (0.0%, 2.0%)	0.0% (0.0%, 1.1%)
	*Enterobacterales*	137 (32.2%)	36 (8.5%)	252 (59.3%)	97.9% (96.0%, 98.9%)	88.5% (85.1%, 91.2%)	11.5% (8.8%, 14.9%)	0.0% (0.0%, 2.7%)	0.0% (0.0%, 1.5%)
	*Acinetobacter*	22 (19.5%)	1 (0.9%)	90 (79.6%)	94.7% (88.9%, 97.5%)	99.1% (95.2%, 99.8%)	0.9% (0.2%, 4.8%)	0.0% (0.0%, 14.9%)	0.0% (0.0%, 4.1%)
	*Pseudomonas aeruginosa*	25 (73.5%)	1 (2.9%)	8 (23.5%)	94.1% (80.9%, 98.4%)	91.2% (77.0%, 97.0%)	8.8% (3.0%, 23.0%)	0.0% (0.0%, 13.3%)	0.0% (0.0%, 32.4%)
	Other negative organisms	not tested	not tested	not tested					
Colistin ^2^	Total	0 (0%)	469 (90.2%)	51 (9.8%)	96.0% (93.9%, 97.3%)	95.8% (93.7%, 97.2%)	4.2% (2.8%, 6.3%)		0.0% (0.0%, 7.0%)
	*Enterobacterales*	0 (0%)	339 (88.1%)	46 (11.9%)	95.6% (93.0%, 97.2%)	95.6% (93.0%, 97.2%)	4.4% (2.8%, 7.0%)		0.0% (0.0%, 7.7%)
	*Acinetobacter*	0 (0%)	106 (95.5%)	5 (4.5%)	96.4% (91.1%, 98.6%)	95.5% (89.9%, 98.1%)	4.5% (1.9%, 10.1%)		0.0% (0.0%, 43.4%)
	*Pseudomonas aeruginosa*	0 (0%)	24 (100%)	0 (0%)	100.0% (86.2%, 100.0%)	100.0% (86.2%, 100.0%)	0.0% (0.0%, 13.8%)		
	Other negative organisms	not tested	not tested	not tested					
Imipenem	Total	313 (60.3%)	5 (1%)	201 (38.7%)	95.8% (93.7%, 97.2%)	97.7% (96.0%, 98.7%)	1.7% (0.9%, 3.3%)	0.6% (0.2%, 2.3%)	0.5% (0.1%, 2.8%)
	*Enterobacterales*	275 (72%)	3 (0.8%)	104 (27.2%)	95.8% (93.3%, 97.4%)	97.9% (95.9%, 98.9%)	1.8% (0.9%, 3.7%)	0.0% (0.0%, 1.4%)	1.0% (0.2%, 5.2%)
	*Acinetobacter*	21 (18.6%)	0 (0%)	92 (81.4%)	97.3% (92.5%, 99.1%)	100.0% (96.7%, 100.0%)	0.0% (0.0%, 3.3%)	0.0% (0.0%, 15.5%)	0.0% (0.0%, 4.0%)
	*Pseudomonas aeruginosa*	17 (70.8%)	2 (8.3%)	5 (20.8%)	87.5% (69.0%, 95.7%)	83.3% (64.1%, 93.3%)	8.3% (2.3%, 25.8%)	11.8% (3.3%, 34.3%)	0.0% (0.0%, 43.4%)
	Other negative organisms	not tested	not tested	not tested					
Levofloxacin	Total	214 (36.3%)	31 (5.3%)	344 (58.4%)	98.1% (96.7%, 99.0%)	89.8% (87.1%, 92.0%)	10.2% (8.0%, 12.9%)	0.0% (0.0%, 1.8%)	0.0% (0.0%, 1.1%)
	*Enterobacterales*	148 (36.5%)	13 (3.2%)	245 (60.3%)	98.3% (96.5%, 99.2%)	90.6% (87.4%, 93.1%)	9.4% (6.9%, 12.6%)	0.0% (0.0%, 2.5%)	0.0% (0.0%, 1.5%)
	*Acinetobacter*	22 (19.3%)	7 (6.1%)	85 (74.6%)	98.2% (93.8%, 99.5%)	91.2% (84.6%, 95.2%)	8.8% (4.8%, 15.4%)	0.0% (0.0%, 14.9%)	0.0% (0.0%, 4.3%)
	*Pseudomonas aeruginosa*	20 (58.8%)	4 (11.8%)	10 (29.4%)	94.1% (80.9%, 98.4%)	85.3% (69.9%, 93.6%)	14.7% (6.4%, 30.1%)	0.0% (0.0%, 16.1%)	0.0% (0.0%, 27.8%)
	Other negative organisms	24 (68.6%)	7 (20%)	4 (11.4%)	100.0% (90.1%, 100.0%)	80.0% (64.1%, 90.0%)	20.0% (10.0%, 35.9%)	0.0% (0.0%, 13.8%)	0.0% (0.0%, 49.0%)
Meropenem	Total	356 (61.3%)	3 (0.5%)	222 (38.2%)	97.8% (96.2%, 98.7%)	98.8% (97.5%, 99.4%)	1.2% (0.6%, 2.5%)	0.0% (0.0%, 1.1%)	0.0% (0.0%, 1.7%)
	*Enterobacterales*	310 (72.3%)	2 (0.5%)	117 (27.3%)	98.6% (97.0%, 99.4%)	99.3% (98.0%, 99.8%)	0.7% (0.2%, 2.0%)	0.0% (0.0%, 1.2%)	0.0% (0.0%, 3.2%)
	*Acinetobacter*	22 (19.1%)	0 (0%)	93 (80.9%)	94.8% (89.1%, 97.6%)	98.3% (93.9%, 99.5%)	1.7% (0.5%, 6.1%)	0.0% (0.0%, 14.9%)	0.0% (0.0%, 4.0%)
	*Pseudomonas aeruginosa*	21 (61.8%)	1 (2.9%)	12 (35.3%)	97.1% (85.1%, 99.5%)	94.1% (80.9%, 98.4%)	5.9% (1.6%, 19.1%)	0.0% (0.0%, 15.5%)	0.0% (0.0%, 24.2%)
	Other negative organisms	3 (100%)	0 (0%)	0 (0%)	100.0% (43.8%, 100.0%)	100.0% (43.8%, 100.0%)	0.0% (0.0%, 56.2%)	0.0% (0.0%, 56.2%)	

^1^ The determination of the SIR results for the MIC values of the experimental product NMIC-461 against ceftazidime and ceftriaxone was based not only on the breakpoints provided in CLSI M100 but also took into consideration the expert rule 1529. However, for the purpose of ensuring comparability with the BMD method, the SIR results for ceftazidime and ceftriaxone in this table were determined solely by referencing the breakpoints in CLSI M100. ^2^ Regarding the SIR for colistin, the breakpoints from CLSI M100 were referenced to re-evaluate the SIR results based on the MIC outcomes, and the consistency of the results was subsequently calculated.

## Data Availability

All data generated or analyzed during this study are included in this published article and its supplementary information files. The raw data supporting the conclusions of this article will be made available by the authors on reasonable request.

## Supplementary Materials

The following supporting information can be downloaded at: https://www.mdpi.com/article/10.3390/antibiotics15030286/s1, Figure S1: Evaluation of AST and CPO; Figure S2: *Enterobacterales* MIC; Figure S3: *Pseudomonas aeruginosa* MIC; Figure S4: *Acinetobacter* spp. MIC; Figure S5: other non-*Enterobacterales* MIC; Table S1: Oligonucleotide sequences of the primers used in this study; Table S2: The MIC of positive unclassified strains for ceftazidime-avibactam, colistin, meropenem, fosfomycin, tigecycline, imipenem, ceftazidime, cefepime, cefoxitin, and aztreonam; Table S3: The raw experimental data for 714 enrolled isolates; Text S1: The inclusion and exclusion criteria of this study.
