# Supplementary material for: Multicenter Analytical Performance Evaluation of the BD Phoenix NMIC-461 Panel for Carbapenemase Classification and Antimicrobial Susceptibility Testing of Enterobacterales, Pseudomonas aeruginosa, and Acinetobacter spp
Source: Antibiotics (Basel). 2026 Mar 12;15(3):286. doi: 10.3390/antibiotics15030286 (PMC13023592; doi:10.3390/antibiotics15030286)
Supplement: Supplementary file 1 [file antibiotics-15-00286-s001.zip › Supplementary Figure S2 Enterobacterales MIC.pdf]

A

	<=2/1	4/2	8/4	16/8	>16/8
>16/8	ME	1		4	154
16/8			19	32	1
8/4	4	29	80	4	
4/2	46	25	2		1
<=2/1	26				VME

B

ME	<=0.25	0.5	1	>1
>1	2	2	3	238
1		2	1	
0.5	4	12	1	
<=0.25	155			VME

C

	<=0.25/4	0.5/4	1/4	2/4	4/4	8/4	>8/4
>8/4	ME 1						61
8/4				1	4	6	
4/4			2	22	7		1
2/4	1	1	8	10			
1/4	6	7	9	2			
0.5/4	79	10	7				
<=0.25/4	176	5	1	1			VME

D

	<=0.5	1	2	4	8	16	>16
>16	ME					3	145
16					3	9	11
8					5	3	
4				7	10	1	1
2		1	1	2			
1	2	7	5	1	1		
<=0.5	165	5	1			1	VME

E

	<=0.5	1	2	4	>4
>4	ME				239
4					1
2					
1					
<=0.5	185	1	1		1

VME

F

	<=0.06	0.12	0.25	0.5	1	2	>2
>2	ME				1	2	232
2					1	8	1
1				10	4		
0.5		5	29	7			
0.25	1	16	10	2			
<=0.125	66	3	2	2		1	
<=0.0625							VME

G

	<=1	2	4	>4
>4				26
4			1	2
2				
<=1	321	18	8	9

H

	<=0.25	0.5	1	2	4	8	>8
>8	ME				1	1	94
8					1	1	3
4					1		
2	1	1	3	2			
1	10	6	7	1	1		1
0.5	58	17	12				
<=0.25	148	8	2				2

VME

I

	<=0.25	0.5	1	2	4	>4
>4	ME			1	11	216
4				3	7	1
2			1	5	1	
1	2	31	8			
0.5	28	16				
<=0.25	67	2	4		2	VME

J

	<=0.25	<=0.5	0.5	1	2	4	8	16	32	>32
>32	ME								4	94
32									3	3
16								2	3	1
8							2	2	1	
4					1	1				
2						1				
1	3		1							
0.5	2									
<=0.5	6									
<=0.25	297				1	1				VME

Figure A-J compares the MIC distributions of Amoxicillin-clavulanate, Ceftaroline, Ceftazidime-avibactam, Ceftazidime, Ceftriaxone, Ciprofloxacin, Colistin, Imipenem, Levofloxacin, and Meropenem against *Enterobacterales*, as determined by two testing methods: BMD and the NMIC-461 panel. The horizontal axis represents the MIC values obtained by the BMD method, while the vertical axis represents the MIC values obtained by the NMIC-461 panel.
