# Supplementary material for: Multicenter Analytical Performance Evaluation of the BD Phoenix NMIC-461 Panel for Carbapenemase Classification and Antimicrobial Susceptibility Testing of Enterobacterales, Pseudomonas aeruginosa, and Acinetobacter spp
Source: Antibiotics (Basel). 2026 Mar 12;15(3):286. doi: 10.3390/antibiotics15030286 (PMC13023592; doi:10.3390/antibiotics15030286)
Supplement: Supplementary file 1 [file antibiotics-15-00286-s001.zip › Supplementary Figure S3 Pseudomonas aeruginosa MIC.pdf]

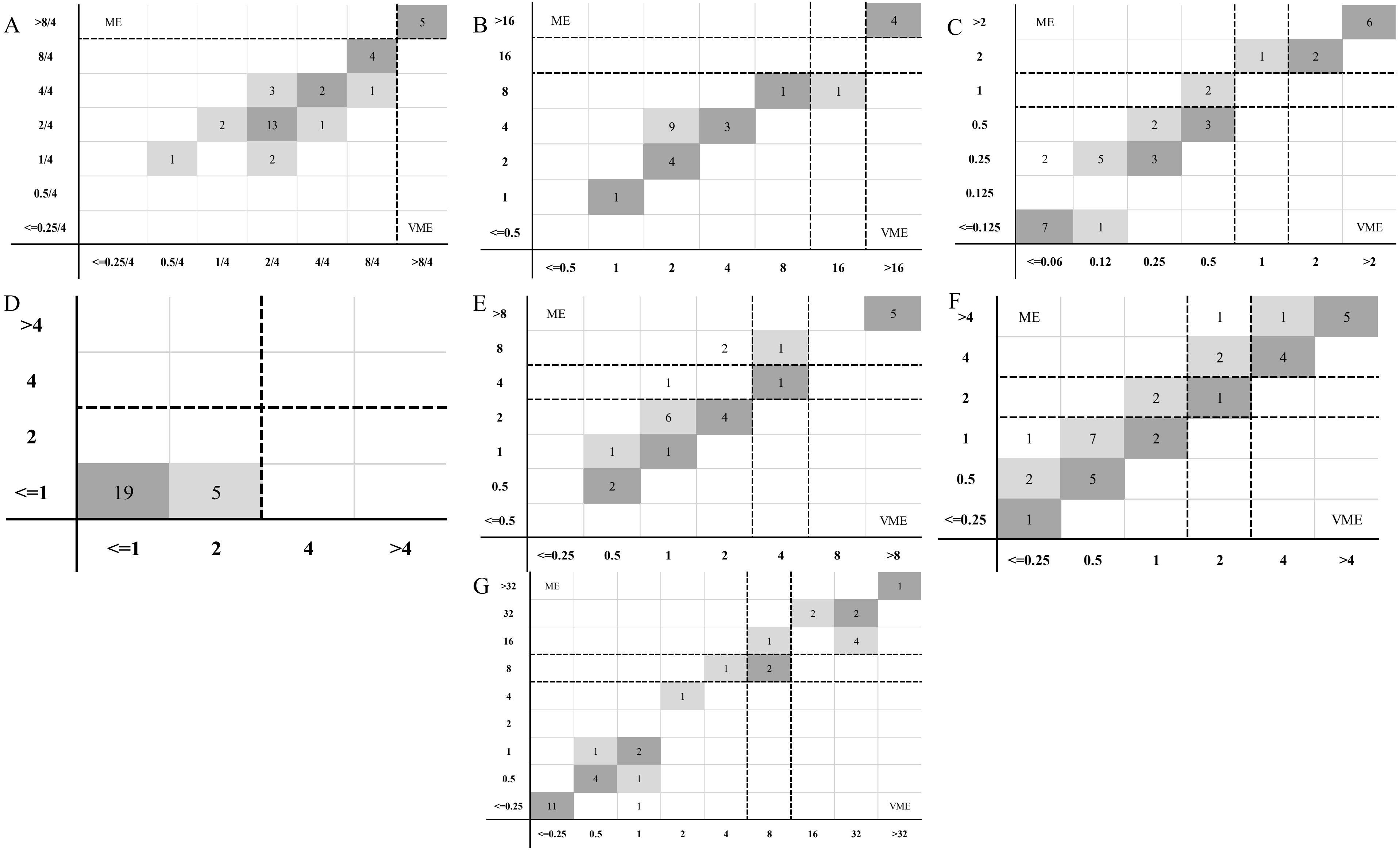

Figure A-G compares the MIC distributions of Ceftazidime-avibactam, Ceftazidime, Ciprofloxacin, Colistin, Imipenem, Levofloxacin, and Meropenem against *Pseudomonas aeruginosa*, as determined by two testing methods: BMD and the NMIC-461 panel. The horizontal axis represents the MIC values obtained by the BMD method, while the vertical axis represents the MIC values obtained by the NMIC-461 panel.
