# Supplementary material for: Multicenter Analytical Performance Evaluation of the BD Phoenix NMIC-461 Panel for Carbapenemase Classification and Antimicrobial Susceptibility Testing of Enterobacterales, Pseudomonas aeruginosa, and Acinetobacter spp
Source: Antibiotics (Basel). 2026 Mar 12;15(3):286. doi: 10.3390/antibiotics15030286 (PMC13023592; doi:10.3390/antibiotics15030286)
Supplement: Supplementary file 1 [file antibiotics-15-00286-s001.zip › Supplementary Table S1.pdf]

Supplementary Table S1. Oligonucleotide sequences of the primers used in this study.

Target gene	Primer name	sequence	Product size (bp)
bal_{KPC}	KPC-F	5'-CGATACCACGTTCCGTCTGGA-3'	409
	KPC-R	5'-GMTGACGCCCAATCCCTAGAGC-3'	
bal_{IMP-4}	IMP-4-F	5'-AAGAAGTTAACGGGTGGGGC-3'	550
	IMP-4-R	5'-GTTTCAAGAGTGATFCGTCTC-3’	
bal_{IMP-8}	IMP-8-F	5'-ACGGTTGGGGTGTTGTTTCT-3'	544
	IMP-8-R	5'-TTCAAGAGTGATGCGTCCCC-3'	
bal_{VIM}	VIM-F	5'-CAGTCTCCACGCACTTTCA-3'	419
	VIM-R	5'-GAGCAAGTCTAGACCGCCCG-3'	
bal_{NDM}	NDM-F	5'-CCAGCAAATGGAAACTGGCG-3'	453
	NDM-R	5'-GGGCCGGGGTAAAATACCTT-3'	
bal_{OXA-48}	OXA-48-F	5'-ATCACAGGGCGTAGTTGTGC-3'	623
	OXA-48-R	5'-AACCATCCGATGTGGGCATA-3'	
bal_{OXA-23}	OXA-23-F	5'-TAATGCTCTAAGCCGCGCAA-3'	616
	OXA-23-R	5'-CAGCTGTTTTAATGATTTCATC-3'	
bal_{OXA-24}	OXA-24-F	5'-TGATGAAGCTCAAACACAGGGT-3'	585
	OXA-24-R	5'-CCATTAGCTTGCTCCACCCA-3'	
bal_{OXA-58}	OXA-58-F	5'-AGTATTGGGGCTTGTGCTGA-3'	599
	OXA-58-R	5'-TCCCCTCTGCGCTCTACATA-3'	

Abbreviations: KPC, Klebsiella pneumoniae carbapenemase; NDM, New Delhi metallo-beta-lactamase; VIM, Verona integron-borne metallo-beta-lactamase; IMP, imipenemase; OXA, oxacillinase; F, Forward; R, Reverse.
