# Supplementary material for: Multicenter Analytical Performance Evaluation of the BD Phoenix NMIC-461 Panel for Carbapenemase Classification and Antimicrobial Susceptibility Testing of Enterobacterales, Pseudomonas aeruginosa, and Acinetobacter spp
Source: Antibiotics (Basel). 2026 Mar 12;15(3):286. doi: 10.3390/antibiotics15030286 (PMC13023592; doi:10.3390/antibiotics15030286)
Supplement: Supplementary file 1 [file antibiotics-15-00286-s001.zip › Supplementary Text S1.pdf]

Supplementary Text S1. The inclusion and exclusion criteria of this study.

Strains that meet the following criteria will be included in the subsequent analysis:

1. Meeting the inclusion criteria and not meeting the exclusion criteria.

a) Inclusion criteria: Clinical strains of Gram-negative aerobic or facultative anaerobic bacteria belonging to the Enterobacteriaceae family or non-Enterobacteriaceae family, whose species types have been confirmed by mass spectrometry identification.

b) Exclusion criteria:

1) Multiple isolates of the same bacterial species from one specimen;

2) Strains of the same bacterial species (at the species level) sourced from the same patient;

3) Bacterial species beyond the identification and antimicrobial susceptibility range of the BD Phoenix^{TM} system;

4) Specimens collected solely for this experiment.

2. Not meeting the exclusion criteria:

1) Colonies with poor growth or no growth;

2) Impure or contaminated strains.

3. Pass the quality control.

4. Have valid antimicrobial susceptibility test results: Both the Minimum Inhibitory Concentration (MIC) and the Susceptible, Intermediate, Resistant (SIR) results of the test panel NMIC-461 and the control method (NMIC-413 or Broth Microdilution, BMD) are valid.

5. Have valid results of the resistance mechanism: Both the results of the test panel NMIC-461 and the control method (NMIC-413 or modified Carbapenem Inactivation Method, mCIM or Polymerase Chain Reaction, PCR) are valid.

6. The entire panel is valid: This includes situations such as abnormalities in positive or negative control wells, incorrect loading of the Antimicrobial Susceptibility Test Indicator (AST Indicator), other reasons for invalidity such as poor/no growth of the strains, and failure of the operator's proficiency assessment, etc.

The numbers of strains whose antimicrobial susceptibility test results and resistance mechanisms did not enter the subsequent analysis, along with the reasons, are summarized as shown in the following table.

Reasons summary of Organism which not included in final analysis.

**Antimicrobial agent**	**Control Method**	**Reason for not included in final analysis**	**# Organism**
**Amoxicillin-Clavulanate**	BMD	Total	263
		Not meeting the inclusion criteria or meeting the exclusion criteria	3
		Meeting the exclusion criteria	2
		Failed to pass the quality control	0
		No valid result of the antimicrobial susceptibility test	239
		The entire panel is invalid	72
**Ceftaroline**	BMD	Total	271
		Not meeting the inclusion criteria or meeting the exclusion criteria	3
		Meeting the exclusion criteria	2
		Failed to pass the quality control	24
		No valid result of the antimicrobial susceptibility test	242
		The entire panel is invalid	72
**Ceftazidime-Avibactam**	BMD	Total	229

		Not meeting the inclusion criteria or meeting the exclusion criteria	3
		Meeting the exclusion criteria	2
		Failed to pass the quality control	0
		No valid result of the antimicrobial susceptibility test	195
		The entire panel is invalid	72
**Ceftazidime**	BMD	Total	130
		Not meeting the inclusion criteria or meeting the exclusion criteria	3
		Meeting the exclusion criteria	2
		Failed to pass the quality control	0
		No valid result of the antimicrobial susceptibility test	129
		The entire panel is invalid	72
**Ceftriaxone**	BMD	Total	260
		Not meeting the inclusion criteria or meeting the exclusion criteria	3
		Meeting the exclusion criteria	2
		Failed to pass the quality control	0
		No valid result of the antimicrobial susceptibility test	236
		The entire panel is invalid	72
**Ciprofloxacin**	BMD	Total	119
		Not meeting the inclusion criteria or meeting the exclusion criteria	3
		Meeting the exclusion criteria	2
		Failed to pass the quality control	0

		No valid result of the antimicrobial susceptibility test	60
		The entire panel is invalid	72
**Colistin**	BMD	Total	171
		Not meeting the inclusion criteria or meeting the exclusion criteria	3
		Meeting the exclusion criteria	2
		Failed to pass the quality control	0
		No valid result of the antimicrobial susceptibility test	170
		The entire panel is invalid	72
**Imipenem**		Total	172
		Not meeting the inclusion criteria or meeting the exclusion criteria	3
		Meeting the exclusion criteria	2
		Failed to pass the quality control	20
		No valid result of the antimicrobial susceptibility test	171
		The entire panel is invalid	72
**Levofloxacin**	BMD	Total	102
		Not meeting the inclusion criteria or meeting the exclusion criteria	3
		Meeting the exclusion criteria	2
		Failed to pass the quality control	0
		No valid result of the antimicrobial susceptibility test	42
		The entire panel is invalid	72
**Meropenem**	BMD	Total	110

		Not meeting the inclusion criteria or meeting the exclusion criteria	3
		Meeting the exclusion criteria	2
		Failed to pass the quality control	0
		No valid result of the antimicrobial susceptibility test	51
		The entire panel is invalid	72
**CPO**	mCIM or PCR	Total	94
		Not meeting the inclusion criteria or meeting the exclusion criteria	3
		Meeting the exclusion criteria	2
		Failed to pass the quality control	0
		No valid result of the resistance mechanism	83
		The entire panel is invalid	8

Note: If a certain strain is involved in two or more reasons for not entering the final analysis, each involved reason will be counted once, and the Total will be counted only once.
