# Supplementary figures and images for: Multicenter Analytical Performance Evaluation of the BD Phoenix NMIC-461 Panel for Carbapenemase Classification and Antimicrobial Susceptibility Testing of Enterobacterales, Pseudomonas aeruginosa, and Acinetobacter spp
Source: Antibiotics (Basel). 2026 Mar 12;15(3):286. doi: 10.3390/antibiotics15030286 (PMC13023592; doi:10.3390/antibiotics15030286)

### Evaluation of AST

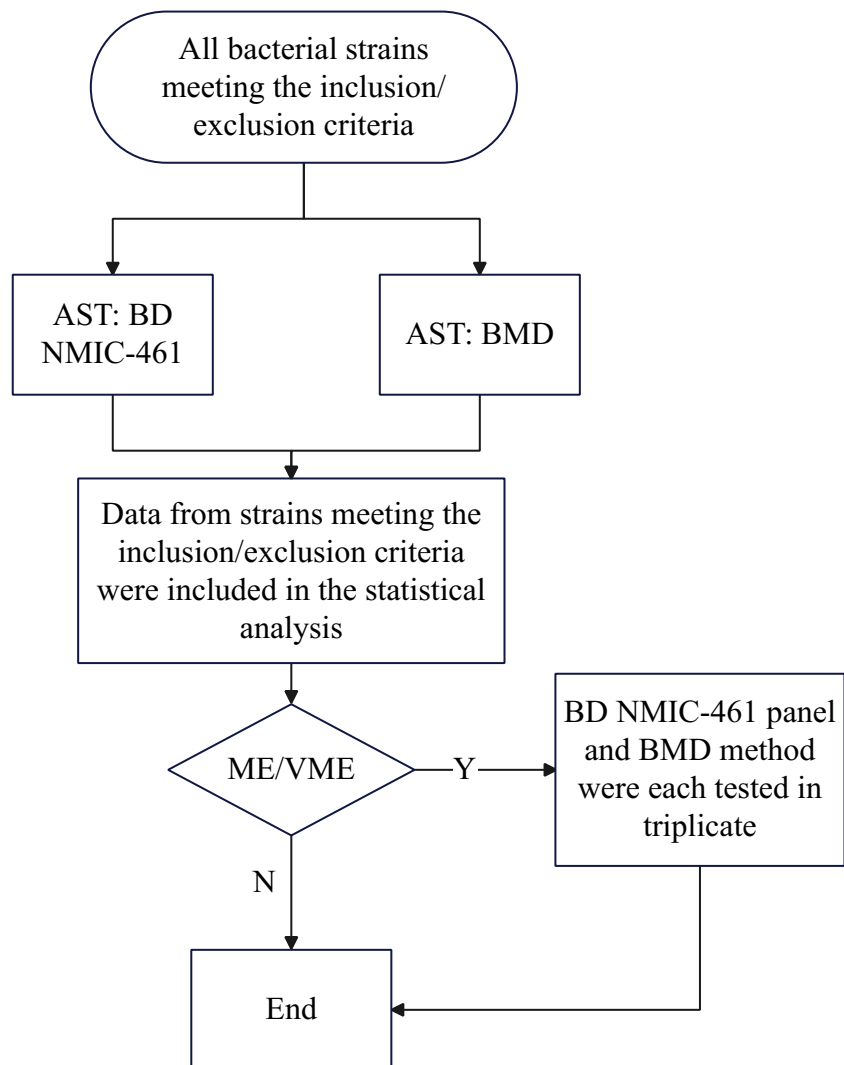

### Evaluation of CPO

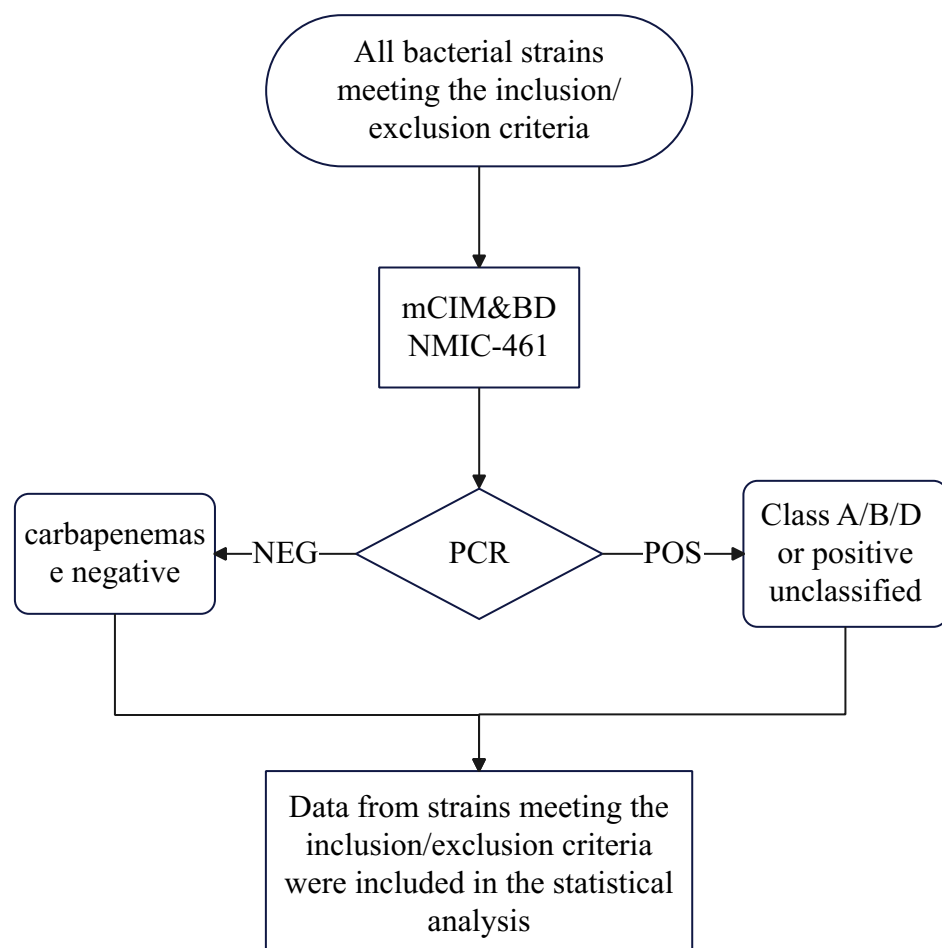

Supplement: Supplementary file 1 [file antibiotics-15-00286-s001.zip › Supplementary Figure S1 Evaluation of AST and CPO.pdf]
